# The Role of Electrocardiographic Markers for Predicting Atrial Fibrillation in Patients with Acute Ischemic Stroke: Data from the BIOSIGNAL Cohort Study

**DOI:** 10.3390/jcm12216830

**Published:** 2023-10-29

**Authors:** Valerie Schütz, Svetlana Dougoud, Katja Bracher, Markus Arnold, Juliane Schweizer, Christos Nakas, Laura P. Westphal, Corinne Inauen, Thomas Pokorny, Firat Duru, Jan Steffel, Andreas Luft, Katharina Spanaus, Ardan Muammer Saguner, Mira Katan

**Affiliations:** 1 Department of Neurology, University Hospital of Zurich, Neuroscience Center Zurich, University of Zurich, 8006 Zürich, Switzerlandkatja.bracher@gmail.com (K.B.); corinne.inauen@usz.ch (C.I.);; 2 Department of Neurology, University Hospital of Tulln, 3430 Tulln an der Donau, Austria; 3 Department of Cardiology, University Heart Center, University Hospital of Zurich, 8006 Zürich, Switzerland; svetlana.dougoud@usz.ch (S.D.); firat.duru@usz.ch (F.D.);; 4 Laboratory of Biometry, University of Thessaly, 382 21 Volos, Greece; christos.nakas@extern.insel.ch; 5 University Institute of Clinical Chemistry, Inselspital, Bern University Hospital, University of Bern, 3012 Bern, Switzerland; 6 Institute of Clinical Chemistry, University Hospital of Zurich, 8006 Zürich, Switzerland; 7 Center for Translational and Experimental Cardiology (CTEC), Department of Cardiology, Zurich University Hospital, University of Zurich, 8952 Schlieren, Switzerland; 8 Department of Neurology, University Hospital and University of Basel, 4031 Basel, Switzerland

**Keywords:** P-wave abnormalities, biomarkers, acute ischemic stroke, stroke etiology, newly diagnosed atrial fibrillation, secondary prevention

## Abstract

Background and Aims: P-wave abnormalities in the 12-lead electrocardiogram (ECG) have been associated with a higher risk of acute ischemic stroke (AIS) as well as atrial fibrillation (AF). This study aimed to assess pre-determined ECG criteria during sinus rhythm in unselected AIS patients and their value for predicting newly diagnosed atrial fibrillation (NDAF) after hospital admission. Methods: P-wave alterations were measured on 12-lead ECG on admission in all consecutively enrolled patients without known AF between October 2014 and 2017. The outcome of interest was NDAF, identified by prolonged electrocardiographic monitoring within one year after the index AIS. Univariable and multivariable logistic regression was applied to assess the magnitude and independence of the association between pre-selected ECG markers and NDAF. The discriminatory accuracy was evaluated with the area under the receiver operating characteristic curve (AUC), and the incremental prognostic value was estimated with the net reclassification index. Results: NDAF was detected in 87 (10%) of 856 patients during a follow-up of 365 days. Out of the pre-selected ECG parameters, advanced interatrial block (aIAB) and PR interval in lead II were independently associated with NDAF in univariable regression analysis. Only aIAB remained a significant predictor in multivariable analysis. Adding aIAB to the best-performing multivariable regression model improved the discriminatory accuracy to predict NDAF from an AUC of 0.78 (95%-CI 0.77–0.80) to 0.81 (95%-CI 0.80–0.83, *p* < 0.001). Conclusion: aIAB is independently and highly associated with NDAF in patients with AIS, has high inter-rater reliability, and therefore may be helpful to refine diagnostic work-up to search for AF in AIS.

**What is new?**

- Advanced interatrial block (aIAB) and PR duration determined on 12-lead surface ECG in lead II are independently associated with newly diagnosed atrial fibrillation in patients with acute ischemic stroke.
- aIAB significantly improved risk stratification beyond established risk factors.
- aIAB is an easily measurable ECG marker and has a high inter-rater reliability.
- Therefore, aIAB may help refine diagnostic work-up to search for atrial fibrillation in patients with acute ischemic stroke.

## 1. Introduction

Atrial fibrillation (AF) represents one of the most common stroke etiologies. Its detection is crucial for further stroke prevention since it requires secondary prevention with oral anticoagulants instead of antiplatelet drugs, such as in patients with carotid stenosis as the underlying stroke etiology. However, the detection of paroxysmal AF remains challenging. Readily accessible biomarkers and clinical parameters on admission, which are associated with newly diagnosed atrial fibrillation (NDAF), would be helpful for a targeted diagnostic work-up in patients with acute ischemic stroke (AIS) in clinical routine.

Several studies, for example, the ARIC study as well as CHS, assessed the predictive value of P-wave changes on electrocardiography (ECG) concerning their association with incident stroke risk, specifically first ever embolic stroke of undetermined source (ESUS) [1]. Other studies assessed the predictive value of advanced interatrial block (aIAB) and P-terminal force in lead V1 (PTFV1), as well as PR interval in lead II, concerning the detection of AF in stroke-free populations with and without heart disease [2,3,4,5,6,7].

For instance, aIAB was assessed in the Bayes registry as a surrogate marker for AF in patients with heart disease [5].

However, to our knowledge, these markers have yet to be prospectively assessed for their ability to predict NDAF in an unselected large, well-characterized Caucasian AIS population. Therefore, the current study aimed to examine ECG markers to help identify patients with a high risk of NDAF up to 365 days after the index AIS.

## 2. Methods

We chose the most established promising ECG markers from the available literature, including PTFV1and PR interval [6,7,8]. Additionally, we selected aIAB as a more recently discovered promising predictive marker due to its association with atrial fibrosis, supraventricular tachyarrhythmia [9], NDAF, incident stroke in the Bayes registry consisting of an elderly stroke-free population with structural heart disease, as well as stroke recurrence in a large Asian ischemic stroke cohort [1,5,10].

### 2.1. Study Design and Patients

The study is registered with ClinicalTrials.gov (URL: https://www.clinicaltrials.gov (accessed on 18 October 2023); Identifier: NCT02274727).

The study, named the BIOSIGNAL study, is a prospective observational multicenter inception cohort study in patients with AISand adheres to the principles of the Declaration of Helsinki. The local ethics committee (Cantonal Ethics Committee Zurich; Ref.Nr.KEK-ZH-Nr.2014-0001) approved the study, and all patients or their welfare legal guardians provided written informed consent. The study data is available upon request, and it follows the Strengthening the Reporting of Observational Studies in Epidemiology (STROBE) guidelines for observational cohort studies [11].

The corresponding author (K.M.) had full access to all the data in the study and took responsibility for its authenticity and correct data analysis.

For this analysis, we only included patients from the University Hospital Zurich, Switzerland, resulting in a potential population of 1166 consecutively admitted patients. We excluded patients with a history of AF, the presence of AF on the first 12-lead ECG on admission, a pacemaker-ECG, or AF within the first 24 h of ECG monitoring. The final analysis included 856 patients. The median age of the enrolled patients was 70 years, and 40% were female.

All participants underwent a standard stroke etiology work-up, including neurovascular ultrasound, a minimum of an additional 24 h Holter-ECG monitoring, and transthoracic echocardiography. Screening for hypercoagulability syndromes or vasculitis was performed at the treating physician’s discretion. Stroke etiology was assessed using the Trial of Org 10172 in Acute Stroke Treatment (TOAST) classification [12]. Additionally, the CHA2DS2-VASc-Score and AS5F-score were evaluated [13,14].

AIS was defined as an acute localized ischemic lesion in the brain that is not attributed to central nervous system infection, demyelinating diseases, tumors, or degenerative neurologic diseases, lasting longer than 24 h, according to the criteria of the World Health Organization [15].

All patients received a neuro CT or MRI and a routine laboratory evaluation on admission. The focal neurological deficit attributed to AIS was quantified by a stroke specialist using the NIHSS. Diffusion-weighted imaging MRI (DWI-MRI) was available in 731 patients (88%). Lesion size was classified into three size categories: small lesion with a volume < 10 cm^3^, medium size lesion with a volume of 10 to 100 cm^3^, and a large lesion with a volume > 100 cm^3^. Demographic data, vascular risk factors, and vital parameters were collected on admission. Comorbidities were measured by the modified Charlson Comorbidity Index for stroke patients (CCI) [16].

### 2.2. ECG Parameters

Digital 12-lead surface ECGs were obtained at baseline with a velocity of 25 mm/s and a depth of 10 mm/mV (notch filter 40 Hz, AC 50 Hz). The ECG parameters were measured manually in a digital format with the “Iconico-ECG-Screen-caliper” (www.iconico.com, accessed on 10 September 2023). The digital caliper was calibrated against the reference pulse. The calibration was performed in mm and converted to ms or mV. ECGs were evaluated by two independent investigators (S.V., D.S.). The calibration was performed using an enlarged ECG of 600% in a PDF file. Both clinical investigators were trained by an experienced cardiologist (S.A.M.) to minimize inter-rater variation; after that, both investigators measured 15% of ECGs simultaneously and separately to assess the inter-rater reliability of these ECG measurements.

**Figure 1:**
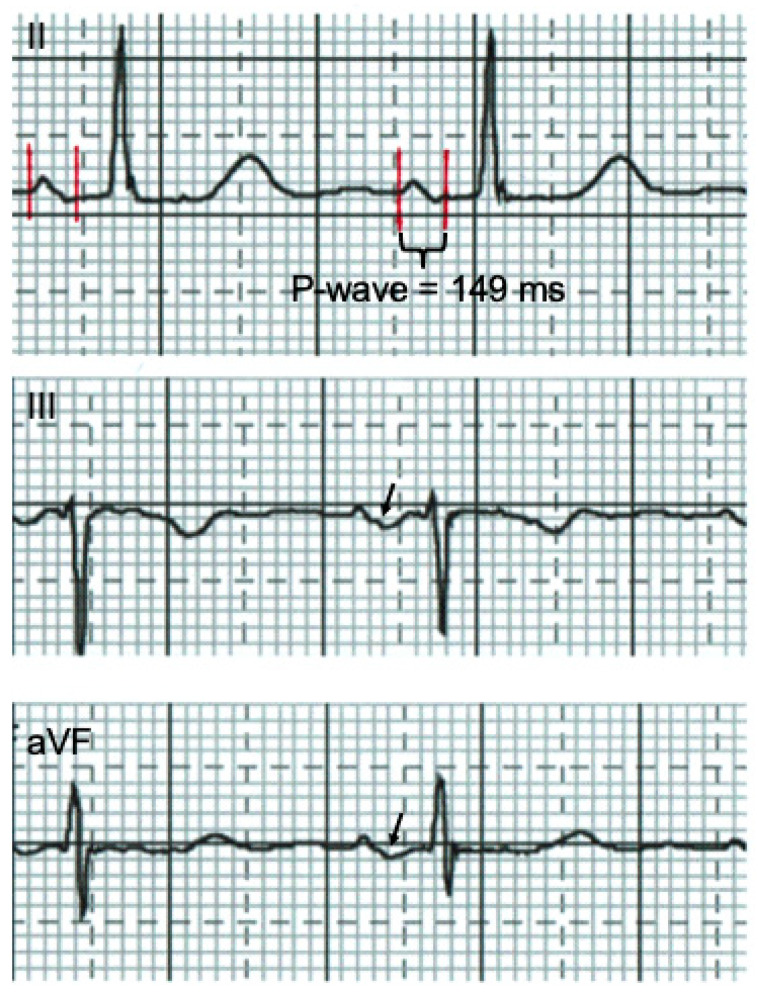
Advanced interatrial block (red lines delineate P wave) with biphasic (arrow) P-wave in II, III, aVF, and a P-wave duration of ≥120 ms in lead II, measured manually with Iconico caliper.

AIAB was defined as a P-wave duration of at least 120 ms in lead II and a biphasic or negative P-wave in lead II, III, and aVF [17] (Figure 1).

PR interval was defined as prolonged if it was longer than 200 ms [18]. PTFV1with a depth of more than 0.1 mV and more than 40 ms was considered abnormal [7].

### 2.3. Echocardiographic Parameters

Echocardiographic parameters were assessed by transthoracic echocardiography from left parasternal, apical, and subcostal windows. Conventional M-mode, 2D, and color Doppler echocardiography were evaluated by experienced investigators using ultrasound transducers with a frequency range between 1 and 5 MHz (Vivid 7 or Vivid E9; GE Vingmed Ultrasound AS, Horten, Norway; iE33; Philips Healthcare, Best, The Netherlands) and according to a standardized clinical protocol. Dimensions and function of cardiac chambers were assessed according to established guidelines for 2D TTE [19]. The left atrial end-systolic diameter (LAESD) was measured from the parasternal long axis at the base of the heart [19].

### 2.4. Biomarker Measurement

Within 24 h of AIS onset, routine blood was drawn in EDTA-containing tubes. Samples were instantly centrifuged at 3000× *g* at 4 °C for 20 min, and plasma was aliquoted and immediately frozen at −80 °C until analysis. Midregional pro-atrial natriuretic peptide (MR-proANP) level (pmol/L) was measured in plasma in a blinded batch analysis by the automated KRYPTOR immunoassay technology (BRAHMS GmbH, Hennigsdorf, Germany) [20].

### 2.5. Outcome Variable

NDAF was defined as AF/atrial tachycardia lasting at least 30 s recorded by any prolonged electrocardiographic monitoring (PCM) during hospitalization after the first 24 h after admission and up to 12 months after AIS. NDAF was diagnosed either during hospitalization, by treating stroke physicians and cardiologists, or after hospital discharge by the general practitioner, cardiologist, or internal medicine specialist after obtaining a 12-lead surface ECG or based on Holter-ECG or other long-term monitoring such as a Reveal event-recorder, according to current guidelines [21].

**Figure 2:**
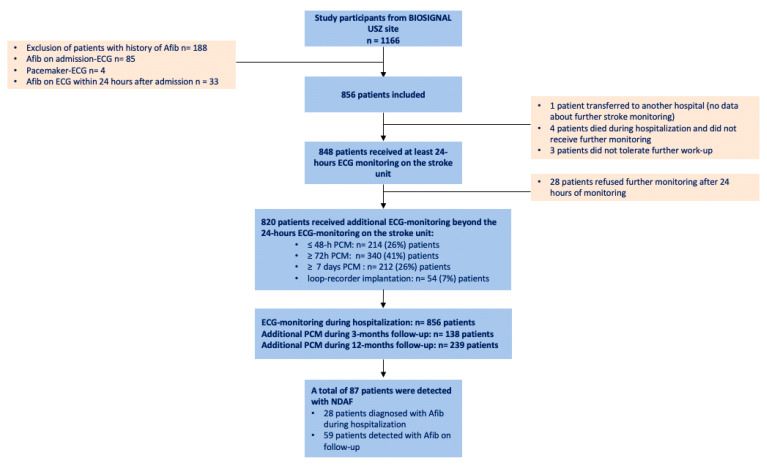
Flow chart of inclusion of the participants of the sub-cohort of the BIOSIGNAL cohort study and additional cardiac monitoring after acute ischemic stroke within 365 days after the index stroke during hospitalization and follow-up.

All patients with AIS obtained at least one continuous 24 h ECG monitoring in the stroke unit. For patients without a history of AF or evidence of AF after the first 24 h of ECG monitoring, at least 48 h of Holter-ECG monitoring was recommended according to the ESO guidelines. Patients without evidence of AF at discharge received further ambulatory PCM, preferably a 7-day Holter-ECG or implantable cardiac devices as the study protocol demands. If patients refused, repeated 48 h Holter-ECG was suggested. (For details, look at Figure 2.)

Follow-up information such as 12-lead ECGs and PCM were obtained 3 and 12 months after the index AIS event during an outpatient visit, or the information about elsewhere performed outpatient PCM was obtained via a structured telephone interview by a stroke physician.

### 2.6. Inter-Rater Reliability of the ECG Markers

To measure the inter-rater reliability, 50 randomly selected ECGs were analyzed by two investigators independently, and the assessment was performed using Cronbach’s alpha.

### 2.7. Statistical Analysis

Discrete variables are presented as counts (percentages) and continuous variables as medians (interquartile ranges [IQRs]). MR-proANP and PTFV1 were transformed by logarithm (base 10). AIAB was defined as a dichotomous variable. We performed the Pearson chi-squared test and the Mann–Whitney U test for continuous non-normally distributed data for two-group comparison for categorical baseline measurements.

We conducted further analysis only with aIAB that was highly significant (*p* < 0.001) in the univariate regression analysis after Bonferroni correction. To assess the magnitude of association with NDAF after the index AIS, as well as to determine the independence of aIAB from known demographic (i.e., age, gender) and vascular risk factors (coronary heart disease, hypertension, smoking, sex, age, obesity, stroke of unknown etiology, LAESD), multivariable logistic regression models were built to calculate odds ratios (OR) and 95% confidence intervals (95% CI).

Two models were generated, including the variables that were highly significant after Bonferroni correction in univariate regression analysis.

We built a parsimonious model (1) excluding variables that require further (more time-consuming and costly) diagnostic work-up and thus health care resources, such as echocardiographic parameters. This parsimonious model 1, including AS5F, logMR-proANP (pmol/L) as well as aIAB, would be especially interesting for healthcare settings with limited access to specialist-dependent diagnostics 24/7, as AS5F is a clinical score and can be assessed easily, including AS5F, logMR-proANP (pmol/L) as well as aIAB, would be especially interesting for healthcare settings with limited diagnostic resources, as AS5F is a clinical score and can be assessed easily [14] and MR-proANP is a valid blood biomarker to detect the risk of NDAF and might be part of the routine stroke work-up in the future [22]. For risk stratification, we elaborated an online risk calculator available at https://aiabndaf.shinyapps.io/dynnomapp/ (accessed on 10 September 2023).

Age was not separately included in the predictive model as the variable AS5F comprises the product of age with factor 0.76 and adding the factor 9 if the NIHSS was less than 5, or factor 21 if the NIHSS was higher than 5: AS5F = (0.76 × age) + (9 × NIHSS ≤ 5) + (21 × NIHSS > 5) [14].

We also built a second model (2) including the selected ECG parameters and all the clinical variables significantly associated with NDAF in the univariate regression analysis after Bonferroni correction, i.e., AS5F, LAESD, large vessel stroke, and MR-proANP. As LAESD presented missing data, we performed multiple imputations with chained equation (MICE) to address missing bias in this model. To handle the different variable types, “predictive mean matching” criteria were applied whenever necessary [23].

To assess the discriminatory ability of the constructed models, receiver operating characteristic (ROC) curves and AUC were calculated for both models with and without the best-performing ECG-marker aIAB, and the likelihood ratio test was employed for comparison. The continuous net reclassification index (NRI) was computed to assess improvement in classification when adding aIAB to models 1 and 2.

To further assess the performance of our predictive model, we performed 10-fold cross-validation with both models, including the ECG marker. The study sample was split into ten equal samples: nine-tenths of the sample was used for training and one-tenth for testing. This process was iterated a thousand times, and the mean of the ORs was calculated.

Furthermore, we calculated sensitivity and specificity measures for unadjusted MR-proANP cut-off levels with different diagnostic thresholds. All statistical analyses were performed using Stata 16.1 (StataCorp LLC, College Station, TX, USA).

## 3. Results

### 3.1. Cohort Characteristics

Out of 1166 enrolled patients, 188 had a history of AF, 85 patients had AF on the first routine 12-lead ECG on admission, and 4 patients had a pacemaker with atrial stimulation. Thirty-three patients had AF on ECG within the first 24 h. Therefore, these patients were excluded from the ECG analysis. A total of 856 patients who presented in sinus rhythm on 12-lead ECG on admission and during the first 24 h remained eligible for the analysis (Figure 2). The median age of the cohort was 70 years (IQR 59–79), and 329 (40%) of them were female.

Overall, 820 (96%) of all eligible patients (n = 856) who survived the first 24 h and were not transferred to another hospital or had an NDAF in the meantime received at least 48 h of total ECG monitoring during hospitalization or follow-up. Additional PCM monitoring during the 3-month follow-up was performed on 138 patients, and additional PCM monitoring on 239 patients out of the 856 included patients during the 12-month follow-up. Overall, 606 (75%) patients received at least 72 h of ECG monitoring, and the median PCM duration was 7 days (IQR 2–9 days) (see Figure 2).

A total of 87 (10%) patients were diagnosed with NDAF (80 patients showed AF, and seven patients had atrial tachycardia lasting at least 30 s) during a follow-up of 365 days. Among these, 28 (32%) were diagnosed with NDAF during hospitalization, and 59 (68%) were diagnosed with NDAF after hospital discharge within one year after the index AIS. Baseline characteristics stratified by the occurrence of NDAF are summarized in Table 1.

Patients with NDAF were older than those without atrial fibrillation; the AS5F was higher in patients with NDAF compared to those without atrial fibrillation detection, as well as LAESD, which had a median of 4.1 cm in patients with NDAF in comparison to 3.8 in the population without atrial fibrillation detection. We provide these parameters between these two groups in Table 1.

In univariable regression analysis, age, AS5F, LAESD, MR-proANP, large vessel stroke (TOAST 1), PR interval, and aIAB were significantly associated with NDAF.

### 3.2. Inter-Rater Reliability for ECG Parameters

The inter-rater reliability (Cronbach’s α) for aIAB was excellent with an α of 0.84 (95%-CI, 0.69–1.00), as well as for PTFV1 with an α of 0.87 (95%-CI 0.56–1.19). However, the PR interval’s inter-rater reliability was moderate, with an α of 0.58 (95%-CI 0.23–0.93). This moderate inter-rater reliability could be due to the occasional bad quality of the ECG due to motion artifacts.

### 3.3. Association of PTFV1 and PR Interval with NDAF

We did not find a significant association of PTFV1 with NDAF in the univariable regression analysis after Bonferroni correction. PR interval was significantly associated with NDAF in univariable regression analysis. However, it did not remain significant in the multivariable models. (See Supplementary Tables S1 and S2)

### 3.4. Association of aIAB with NDAF

The presence of aIAB was more frequent in patients developing NDAF during follow-up (60% vs. 25% in the group without NDAF (Table 1)). In univariable regression analysis, aIAB was significantly associated with NDAF and the strongest ECG predictor of NDAF (OR 4.45, 95%-CI 2.78–7.12; *p* < 0.001).

We built a parsimonious multivariable prognostic model 1, which comprised only readily accessible parameters in the first hours after stroke (aIAB, MR-proANP, and the clinical AS5F score). In this model, the magnitude of the association of aIAB did not significantly change with an OR of 3.71 (95% CI 2.29–6.00; *p* < 0.001). This was also true for the multivariable model 2, including aIAB and all clinical variables significantly associated with NDAF in the univariable logistic regression, namely AS5F, LAESD, MR-proANP, and large vessel stroke. In this multivariable model, the magnitude of the association of aIAB with NDAF remained stable with multiple imputations (OR of 3.81, 95%-CI 2.33–6.23).

Adding aIAB improved the discriminatory accuracy of model 1 for prediction of NDAF from an AUC of 0.69 (95%-CI 0.63–0.75) to 0.76 (95%-CI 0.71–0.81), (NRI 0.69, *p* < 0.001) as well as for an AUC of 0.73 (95%-CI 0.68–0.79) after 10-fold cross-validation.

Adding aIAB to model 2 to predict NDAF improved the accuracy from an AUC of 0.78 (95% CI 0.77–0.80) to 0.81 (95% CI 0.80–0.83, *p* < 0.001), (NRI 0.66, *p* < 0.001). After performing 10-fold cross-validation, the AUC in model 2 was 0.82 (95%-CI 0.80–0.83), adding aIAB. (See Table 2)

### 3.5. Association of MR-proANP and LAESD with NDAF

We found a significant association of MR-proANP with NDAF in the univariable and the multivariable regression model (Table 2 and Table 3). LAESD was also significantly associated with NDAF in the univariable regression analysis yet did not reach sufficient significance in the multivariable model, probably due to many missing data, even after multiple imputations (Table 3). MR-proANP was significantly higher in the group of NDAF (Table 1). We measured the sensitivity and specificity of established MR-proANP cut-offs to detect NDAF [22]. MR-proANP levels of ≥255pmol/L showed a specificity of 89.14%. The different cut-off values are provided in Table 4.

## 4. Discussion

Electrocardiographic P-wave abnormalities are associated with a higher cardioembolic stroke risk and are considered a marker of atrial cardiopathy, paroxysmal AF, and an increased risk of stroke. Yet, their predictive role for detecting newly diagnosed paroxysmal AF in patients after AIS is not well defined. To our knowledge, this is the first study to assess the association of electrocardiographic P-wave abnormalities systematically and prospectively in an unselected large cohort of patients with AIS and NDAF during follow-up. The 12-lead ECG is an inexpensive and easily accessible tool that may improve the identification of AF in stroke patients. Since the detection of AF during follow-up after AIS can be challenging, expensive (e.g., implantation of implantable loop recorders), and time-consuming, simple 12-lead ECG parameters on hospital admission may help to select patients, which may benefit most from prolonged ECG monitoring, especially in regions with limited health care resources.

Our study has the following main findings:

1. NDAF was detected in 10% during follow-up after the index AIS. This finding is similar to other studies in the field, such as Find AF and CRYSTAL AF, with 5–12% within one year of follow-up, depending on the electrocardiographic monitoring method [24,25]. However, Find AF did not include patients with severe ipsilateral carotid or intracranial artery stenosis, and Crystal AF only included cryptogenic strokes compared to our cohort of unselected stroke patients, suggesting that the rate of NDAF is likely independent of initial stroke etiology.
2. The presence of aIAB, an easy-to-measure and robust 12-lead ECG parameter reflecting atrial electrical activation delay, performed best from all electrocardiographic P-wave markers and was independently associated with NDAF in multivariable analysis. Adding aIAB to the regression model, including known risk factors and LAESD, improved the discriminatory accuracy of the model to predict NDAF.

### 4.1. Previous Literature on aIAB

The normal transit time for electrical impulses generated in the sinus node to be conducted throughout the right and left atrium (RA and LA) is less than 110 ms, reflected in the P-wave duration on the surface ECG. AIAB is defined as prolonged conduction time between the RA and LA due to impulse delay or blockage, probably most often in the Bachmann bundle. It is simple to assess and characterized by a prolonged P-wave duration of ≥120 ms in lead II, as well as a biphasic or negative P-wave in the peripheral leads II, III, and aVF (Figure 1). AIAB has been linked to atrial fibrosis and atrial enlargement. This has been confirmed in cardiac imaging studies, including echocardiography and cardiac magnetic resonance imaging. More importantly, aIAB has been previously associated with atrial tachyarrhythmias [1,26], even without documented paroxysmal AF, and may be a good marker for left atrial myopathy and atrial thromboembolism [27].

Indeed, we could show in this large cohort of unselected AIS patients that aIAB measured during sinus rhythm on 12-lead ECG on admission was independently associated with NDAF during follow-up, adding incremental value to existing markers to predict NDAF. Our findings are consistent with the results of other studies [1], and we confirmed these findings in an unselected prospective well-characterized stroke cohort with a higher statistical power.

### 4.2. Previous Literature on PR Interval and PTFV1

In this study, we also analyzed other P-wave abnormalities, such as PTFV1, but we did not find a significant association with NDAF. As demonstrated in previous studies, the missing association of PTFV1 with NDAF might be due to atrial cardiopathy with atrial remodeling and fibrosis without atrial dysrhythmia [28,29].

It has also been shown in a previously conducted study that the PTFV1 amplitude decreased in left atrial fibrotic cardiopathy due to the lack of conductive characteristics of fibrotic tissue, which might also explain the lack of association [30]. The inter-rater reliability was not optimal for the PR interval, which might explain part of the low association. This suggests that these other markers might be less reliable in clinical routine. Moderate inter-rater reliability could be due to the occasional insufficient quality of the ECG due to motion artifacts since it may be difficult to obtain a clean, noiseless ECG in some patients with AIS. Another reason for the missing association with NDAF might be related to the different characteristics of study populations in previously published studies, such as the ARIC study, CHS, or MESA, and the other devices and algorithms to measure these markers [4,31]. Several studies investigating the role of PTFV1 and PR interval in detecting atrial myopathy and AF have used automated measurements. In contrast, we used high-resolution manual measurements of these ECG parameters [4].

### 4.3. Manual Measurement of P-Wave Indices in the Era of Artificial Intelligence (AI)

We performed manual measurements of the ECGs in an era where AI is rising and improving performance. A previous study on assessing the risk of AF by an AI-ECG algorithm has shown excellent performance with a high AUC, sensitivity specificity, and accuracy. The AUC for a single ECG measured by artificial intelligence (AI) was 0.87 (95% CI 0.86–0.88) and 0.90 (95% CI 0.90–0.91), including all ECGs performed on each patient [32]. However, we could also show a significant improvement in the AUC from 0.78 (95% CI 0.86–0.88) to 0.81 (95% CI 0.80–0.83), adding aIAB and an AUC of 0.79 (95% CI 0.77–0.80) to 0.81 (95% CI 0.80–0.83) adding aIAB of one analyzed 12-lead ECG to our models, using a manual measurement method, and thus providing an easy-access and affordable tool for many hospitals with limited financial resources.

### 4.4. Clinical Consequences of Detecting AF in Patients with AIS during Follow-Up

The clinical impact of very short-lasting AF (e.g., <5–6 min) assessed by loop recorders is highly debated, and the evidence for prescribing oral anticoagulation in this population is controversial. Yet, in the most recent guidelines on the detection and treatment of AF, single-lead documentation of AF confirmed by a physician is accepted to diagnose AF [21] and should trigger initiation of oral anticoagulation in this population at high risk for recurrent ischemic stroke. On the other hand, AF detected by Holter-ECG monitoring rather than by loop recorders may represent patients with a higher AF burden more prone to stroke recurrence [33,34].

This study showed highly significant and independent associations between aIAB and NDAF using several statistical methods, including internal cross-validation. The strength of the present study lies in the large cohort size consisting of unselected stroke patients that were prospectively and consecutively included, which contributes to an increased predictive and discriminatory validity of the findings. AIAB (mainly due to its high inter-rater reliability) may be used as a simple and easily accessible tool to refine diagnostic work-up and better distribute available resources for the search of AF. Since, in our study, NDAF was defined as any episode of AF or AT lasting >30 s, we also had 7 patients (out of a total of 87 with NDAF) with AT during PCM on follow-up. The pathophysiology of both might differ to some extent. Probably not all AF detected after stroke can be considered the same (depends on duration, number of occurrences, and methods detected); it might be that some short-lasting episodes also are transient phenomena, not associated with the same stroke recurrence risk and maybe, and therefore should not be treated the same way [35]. However, we do not propose to start oral anticoagulation based on the presence of an aIAB but rather to intensify the search for AF. Other studies are needed to better understand which “type” of AF detected after a stroke needs oral anticoagulation or if we need to ultimately detect AF to decide upon OAC for secondary stroke prevention.

### 4.5. Previous Literature on MR-proANP

Previous studies have shown that serum MR-proANP was linked independently to cardioembolic stroke, known as AF, and NDAF [36]. MR-proANP was also related to a higher risk of cardioembolic stroke among populations without prior stroke events [37]. MR-proANP is mainly secreted due to dilatation of the atria and showed a correlation with the enlargement of the left atrium in a sub-study of the ROMICAT trial [38]. Furthermore, MR-proANP has been proven in prior studies, e.g., the Framingham Heart Study, to be more accurate in predicting atrial dysfunction leading to AF than NT-proBNP [39]. In our research, we were able to show that higher MR-proANP serum levels are also an independent predictor of NDAF during follow-up in patients presenting with AIS. Therefore, in combination with aIAB and other predictive markers of NDAF, such as advanced age, MR-proANP could help to identify patients at high risk of paroxysmal AF.

## 5. Limitations

One important limitation of this study is the lack of an external validation cohort and the single-center design of the study. Therefore, an external validation should also assess the predictive value of aIAB to prove its significant association, as we cannot rule out a center bias.

A limitation could be a potential underdiagnosis of AF/atrial tachycardia during follow-up, as not all patients underwent monitoring by an implantable loop recorder, but most underwent repeated Holter-ECG or R-tests. However, the hereby introduced bias is more likely towards the null hypothesis. Thus, the true association is expected to be even more substantial than the observed one. Also, 75% of patients received at least 72 h of ECG monitoring, representing much more than routinely performed [40]. Echocardiographic data, particularly on LAESD, was not available in many patients. Although increased LAESD was a predictor of NDAF in the univariable regression model, it is possible that a significant association was missed in the multivariable model. Moreover, left atrial volume (LAVI) was shown to have a better predictive value than LAESD [41]. However, LAVI is more challenging to evaluate and, therefore, was not measured routinely in our study. Further studies, such as larger multicenter studies and a validation cohort, are needed to assess the impact of aIAB and echocardiographic parameters to improve strategies to detect NDAF after AIS.

## 6. Conclusions

The presence of aIAB assessed by 12-lead surface ECG during sinus rhythm is independently and highly associated with NDAF in patients presenting with AIS, has a high inter-rater reliability, and therefore can be used as a screening tool to refine diagnostic work-up to search for AF in this population. Thus, aIAB improves the identification of patients with a higher risk of NDAF and thus could contribute to an improved allocation of health care resources. Not in every country can one afford to implant loop recorders in all patients, but in patients with a high risk of NDAF, one could intensify the search, whereas, in patients with a low risk, one could stop the search after the recommended 72 h of monitoring.

## Figures and Tables

**Table 1:** Baseline characteristics stratified by the occurrence of NDAF within one year of follow-up.

	Total	No Atrial Fibrillation	NDAF	*p*-Value **
**No. (%)**	856	769	87	
Demographic data				
Age, median (IQR)	70 (59–80)	69 (57–79)	77 (69–84)	<0.001 **
Female sex, n (%)	345 (40%)	299 (39%)	46 (53%)	0.012
Medical history				
Hypertension, n (%)	593 (69%)	531 (69%)	62 (71%)	0.67
Smoking, n (%)	249 (29%)	231 (30%)	18 (21%)	0.074
Diabetes mellitus, n (%)	124 (14%)	114 (15%)	10 (11%)	0.40
Alcohol abuse, n (%)	54 (6%)	51 (7%)	3 (4%)	0.26
Coronary heart disease, n (%)	160 (19%)	145 (19%)	15 (17%)	0.71
Cardiac heart failure, n (%)	24 (3%)	20 (3%)	4 (5%)	0.28
Dyslipidemia, n (%)	621 (73%)	555 (72%)	66 (76%)	0.46
Family history of CV disease, n (%)	122 (15%)	109 (15%)	13 (16%)	0.74
BMI > 30, n (%)	127 (15%)	112 (15%)	15 (18%)	0.50
Previous stroke/TIA, n (%)	121 (14%)	111 (14%)	10 (11%)	0.46
Peripheral vascular disease, n (%)	125 (15%)	115 (15%)	10 (11%)	0.39
Stroke severity, n (%)				
Mild stroke (NIHSS ≤ 8)	595 (70%)	537 (70%)	58 (67%)	0.54
Moderate stroke (NIHSS 9–15)	161 (19%)	146 (19%)	15 (17%)	0.69
Severe stroke (NIHSS ≥ 16)	100 (12%)	86 (11%)	14 (16%)	0.18
Stroke size (DWI), n (%) *				
Large Lesion	93 (12%)	81 (12%)	12 (16%)	0.31
Medium Lesion	309 (41%)	274 (40%)	35 (47%)	0.30
Small Lesion	350 (47%)	322 (48%)	28 (37%)	0.092
Etiology (TOAST), n (%)				
Large artery atherosclerosis	166 (19%)	162 (21%)	4 (5%)	<0.001 **
Cardioembolism †	103 (12%)	79 (10%)	24 (28%)	0.31
Small vessel disease	123 (14%)	119 (15%)	4 (5%)	0.006
Other etiology	76 (9%)	73 (9%)	3 (3%)	0.060
Unknown etiology	389 (45%)	337 (44%)	52 (60%)	<0.005
Scores				
AS5F	67.5 (58.0–75.9)	67.1 (57.3–75.4)	73.6 (66.0–79.4)	<0.001 **
CHADS-VASc-Score, median (IQR)	2.0 (1.0–3.0)	2.0 (1.0–3.0)	2.0 (1.0–2.0)	0.86
ECG-Markers				
P-terminal force in V1 (µVxms), median (IQR) *	3354 (2135–5015)	3314 (2108–5006)	3728 (2256–5166)	0.29
logP-terminal force in V1, median (IQR)	3.5 (3.3–3.7)	3.5 (3.3–3.7)	3.6 (3.4–3.7)	0.29
PR interval, median (IQR)	178 (162–198)	177 (161–195)	191 (175–212)	<0.001 **
Advanced interatrial block, n (%) *	222 (29%)	172 (25%)	50 (60%)	<0.001 **
Echocardiographic parameters				
LAESD (cm), median (IQR) *	3.8 (3.4–4.2)	3.8 (3.3–4.1)	4.1 (3.7–4.5)	<0.001 **
LVEF in %, median (IQR)	60 (56–64)	60 (56–64)	60 (56–63)	0.59
**Laboratory values, median (IQR)**				
MR-proANP (pmol/L)	110.5 (70.4–182.6)	106.5 (68.2–172.9)	176.4 (106.2–262.6)	<0.001 **

Missing values: * Neuro MRI-DWI data was available in 87.7% of cases; LAESD was missing in 32% (n = 267) of the patients; aIAB was missing in 9.23% (n = 79) of cases; PTFV1 was missing in 11.08% (n = 92); † refers to all patients with cardioembolic etiology classified at baseline due to other causes than AF. Statistics: values are median (IQR) or counts (percentages). Testing was performed using the Mann–Whitney U test for continuous variables and Pearson’s chi-square exact test for binary variables Sixty-three patients were diagnosed with AF/atrial tachycardia during hospitalization or follow-up, but after the first ECG on admission showed sinus rhythm. ** Six variables remained significant in univariable regression analysis after the Bonferroni correction.

**Table 2:** Models including aIAB for the prediction of NDAF before and after multiple imputations.

Univariate Analysis		
Variables	OR	95%-CI
aIAB (binary variable)	4.45	2.78–7.12
**Model 1**		
aIAB (binary variable)	3.71	2.29–6.00
AS5F per points	1.01	0.99–1.04
logMR-proANP (pmol/L)	4.69	1.92–11.50
**Model 2 ‡**		
aIAB (binary variable)	3.81	2.33–6.23
AS5F per points	1.02	1.00–1.04
logMR-proANP (pmol/L)	3.99	1.57–10.15
LAESD per cm	1.63	1.06–2.50
Large vessel stroke	0.14	0.05–0.40

Model 1 (parsimonious model): log MR-proANP, AS5F, and aIAB. ‡ Model 2 (all clinical predictors included with a *p* < 0.001 in the univariate regression analysis after Bonferroni correction, i.e., AS5F, MR-proANP, stroke of unknown etiology, aIAB, and additionally, left atrial end-systolic diameter (LAESD) as it is highly correlated with atrial enlargement); we performed multiple imputation due to missing data in the echocardiographic parameter.

**Table 3:** AUC and NRI for NDAF without and with aIAB, before and after multiple imputation (model 2) or cross-validation.

Predictors	AUC	CI 95%	*p*-Value (LR-TEST)	cNRI
Model 1 **without** aIAB	0.69	(0.63–0.75)	-	-
Model 1 + aIAB	0.76	(0.71–0.81)	***	0.69 ***
Model 2 ‡ **without** aIAB	0.78	(0.77–0.80)	-	-
Model 2 ‡ + aIAB	0.81	(0.80–0.83)	***	0.66 ***
Model 1 + aIAB	0.73	(0.68–0.79)	-	-
Model 2 ‡ + aIAB	**0.82**	(0.80–0.83)	-	-

**Model Improvement:** Indicates significant improvement in AUC compared to the corresponding model without aIAB measured by the likelihood ratio test. cNRI—continuous net reclassification index. **Significance levels**: *** = *p* < 0.001, ‡ Model 2 was imputed due to missing data in LAESD.

**Table 4:** Accuracy measures of MR-proANP for newly diagnosed AF during follow-up.

MR-proANP Cut Point	Sensitivity	Specificity	CC	LR +	LR −
≥156 pmol/L	56.32%	70.89%	69.40%	1.93	0.61
≥200 pmol/L	42.53%	81.46%	77.49%	2.29	0.71
≥255 pmol/L	26.44%	89.16%	82.77%	2.44	0.82

Accuracy measures of MR-proANP for a new diagnosis of AF on follow-up for cut-off values with specificity thresholds of approx. 70, 80, and 90%. CC = correctly classified, LR + = positive likelihood ratio, LR − = negative likelihood ratio.

## Data Availability

Research data regarding this study is available (A.M.S., M.K.) upon reasonable request.

## Supplementary Materials

The following supporting information can be downloaded at: https://www.mdpi.com/article/10.3390/jcm12216830/s1, Table S1: Multivariable regression models with PR-Interval lead II; Table S2: Univariable regression analysis with logP-terminal force as dependent variable and NDAF as independent variable; Table S3: STROBE Statement—checklist of items that should be included in reports of observational studies.
