# Supplementary material for: The Role of Electrocardiographic Markers for Predicting Atrial Fibrillation in Patients with Acute Ischemic Stroke: Data from the BIOSIGNAL Cohort Study
Source: J Clin Med. 2023 Oct 29;12(21):6830. doi: 10.3390/jcm12216830 (PMC10649302; doi:10.3390/jcm12216830)
Supplement: Supplementary file 1 [file jcm-12-06830-s001.zip › jcm-2639139-supplementary.pdf]

Supplements

The data presented in this study are available on request from the corresponding author. The data are not publicly available due to ethical reasons.

Table S1: multivariable regression models with PR-Interval lead II

	**Univariable Analysis**	
**Variables**	**OR**	**95%-CI**
PR-Interval lead II (ms)	1.01	1.00 – 1.01
	**Adjusted OR Model 1**	
PR-Interval lead II (ms)	1.00	1.00 – 1.04
AS5F per points	1.02	1.00 – 1.04
**log**MR-proANP (pmol/l)	6.63	2.67 – 16.42
	**Adjusted OR Model 2‡**	
PR-Interval lead II (ms)	1.00	1.00 – 1.01
AS5F per points	1.01	1.00 – 1.04
**log**MR-proANP (pmol/l)	5.70	2.28 – 14.25
LAESD per cm	1.54	1.01 – 2.35
Large vessel stroke	0.15	0.05 – 0.41

‡ Due to missing data, we performed multiple Imputation

Table S2: univariable regression analysis with logP-terminal force as dependent variable and NDAF as independent variable. logPTFV1 did not show a significant association with NDAF in univariable regression analysis with a narrow CI, and therefore no further analysis was performed.

Univariable Analysis		
**Variable**	**OR**	**95%-CI**
logP-terminal force in lead V1 (µV x ms)	1.00	1.00 – 1.00

Table S3
STROBE Statement—checklist of items that should be included in reports of observational studies

	Item No	Recommendation	Page No
**Title and abstract**	1	(*a*) Indicate the study's design with a commonly used term in the title or the abstract	1
		(*b*) Provide in the abstract an informative and balanced summary of what was done and what was found	1
**Introduction**			
Background/rationale	2	Explain the scientific background and rationale for the investigation being reported	1, 2
Objectives	3	State specific objectives, including any prespecified hypotheses	2
**Methods**			
Study design	4	Present key elements of study design early in the paper	2
Setting	5	Describe the setting, locations, and relevant dates, including periods of recruitment, exposure, follow-up, and data collection	2-5
Participants	6	(*a*) *Cohort study*—Give the eligibility criteria, and the sources and methods of selection of participants. Describe methods of follow-up<br>*Case-control study*—Give the eligibility criteria, and the sources and methods of case ascertainment and control selection. Give the rationale for the choice of cases and controls<br>*Cross-sectional study*—Give the eligibility criteria, and the sources and methods of selection of participants	2-6
		(*b*) *Cohort study*—For matched studies, give matching criteria and number of exposed and unexposed<br>*Case-control study*—For matched studies, give matching criteria and the number of controls per case	N/A
Variables	7	Clearly define all outcomes, exposures, predictors, potential confounders, and effect modifiers. Give diagnostic criteria, if applicable	2 - 10
Data sources/ measurement	8*	For each variable of interest, give sources of data and details of methods of assessment (measurement). Describe comparability of assessment methods if there is more than one group	2 - 10

Bias	9	Describe any efforts to address potential sources of bias	9
Study size	10	Explain how the study size was arrived at	5, 7
Quantitative variables	11	Explain how quantitative variables were handled in the analyses. If applicable, describe which groupings were chosen and why	6
Statistical methods	12	(*a*) Describe all statistical methods, including those used to control for confounding	2 - 7
		(*b*) Describe any methods used to examine subgroups and interactions	N/A
		(*c*) Explain how missing data were addressed	1, 6
		(*d*) *Cohort study*—If applicable, explain how loss to follow-up was addressed<br>*Case-control study*—If applicable, explain how matching of cases and controls was addressed<br>*Cross-sectional study*—If applicable, describe analytical methods taking account of sampling strategy	N/A
		(*e*) Describe any sensitivity analyses	1, 6, 7
